# Society Meetings

**Published:** 1909-07

**Authors:** 


### SOCIETY MEETINGS.

---

The Medical Society of the County of Erie held its regular meeting at the University Club Monday evening, June 21, 1909, at 8 o'clock. under the presidency of Charles A. Wall. Program: Relation of local skin lesion to flat foot. Wm. H. Billings; Nervous sequelæ on infectious diseases, J. W. Putnam; Surgical importance of the vestibulary apparatus, Clayton Brown; Colon bacillaria, Myrtle L. Massey; Localised tuberculosis, James A McLeod.

